# Research Article Quality of life after laparoscopic hysterectomy versus abdominal hysterectomy

**DOI:** 10.1186/s12905-021-01364-8

**Published:** 2021-05-22

**Authors:** Yasushi Kotani, Kosuke Murakami, Risa Fujishima, Akiko Kanto, Hisamitsu Takaya, Masao Shimaoka, Hidekatsu Nakai, Noriomi Matsumura

**Affiliations:** Department of Obstetrics and Gynecology, Kindai University Faculty of Medicine, 377-2 Ohno-higashi, Osaka-sayama, Osaka 589-8511 Japan

**Keywords:** Laparoscopic hysterectomy, Quality of life, 36-Item Short Form Survey (SF-36)

## Abstract

**Background:**

Laparoscopic surgery has been described as a minimally invasive surgery. The purpose of this study is to clarify its minimal invasive features using a patient questionnaire on the postoperative quality of life (QOL) over various time periods following either laparoscopic hysterectomy (LH) or abdominal hysterectomy (AH) and to compare the results.

**Methods:**

This study enrolled 28 patients who underwent total hysterectomy for uterine fibroids in 2012 (14 AH cases and 24 LH cases) were enrolled in this study. The 36-Item Short Form Survey (SF-36) questionnaire was completed on postsurgical day 3; weeks 1, 2, and 4; and month 6. The results were compared between the two groups.

**Results:**

Patients who underwent LH scored significantly higher on physical functioning on postoperative day 3 and week 2; physical role and bodily pain on day 3 and week 1; general health on postoperative day 3, weeks 1, 2, and 4, and month 6; social functioning on day 3; and emotional role on day 3 and week 1. No significant differences were found between vitality and mental health at any time point or in the categories above at any other time point.

**Conclusions:**

Postoperative QOL in LH cases was improved on day 3 and week 1; however, no significant differences between the LH and AH groups were found in most categories at week 4 and month 6. LH leads to superior short-term QOL early in the postoperative period relative to AH.

## Background

Uterine fibroids are a typical benign gynecological disease in sexually mature women, and total hysterectomy is the treatment of choice for symptomatic patients. Although the size of uterine fibroids influences the surgical choice, laparoscopic hysterectomy (LH) is less invasive than abdominal hysterectomy (AH) and is becoming increasingly popular despite limitations in its application dependent on lesion size [1, 2].

Laparoscopic surgery is reported to be less susceptible to surgical blood loss or wound infection and facilitates a shorter hospital stay and faster return to society compared with abdominal surgery [1, 2]. However, these results are based on short-term perioperative studies, which raises the question of whether laparoscopy is truly less invasive than open surgery.

We considered that although laparoscopy unequivocally yields smaller surface wounds, it may be more accurately classified as a minimal access—rather than minimally invasive—procedure given that the degree of intra-abdominal maneuvering is similar to that required for open surgery. Likewise, a question regarding the nature of invasiveness is raised, that is, whether it is minimally invasive in the long term as well as in the short term. Few surveys have been published to date on quality of life (QOL) and satisfaction with laparoscopic and open surgeries.

To our knowledge, no studies to date have investigated the QOL of patients with benign gynecological diseases over short or long postoperative periods using a measure such as the Short Form 36-Item Health Survey (SF-36). In the present study, we compared self-reported health-related postoperative QOL between patients who underwent LH or AH for uterine fibroids. The SF-36 was used to gather self-reported data on patients’ postoperative QOL in the short and long terms to investigate whether laparoscopic surgery is truly a minimally invasive procedure that meaningfully improves QOL.

## Methods

This retrospective study was conducted at Kindai University Hospital and Kindai University Nara Hospital, with the approval of the Ethics Committee. Written informed consent was obtained from all participating respondents (R24-036, R24-058). Eligibility criteria comprised patients diagnosed with uterine fibroids and adenomyosis on preoperative magnetic resonance imaging (MRI) who underwent total hysterectomy. Exclusion criteria comprised those who had underwent LH or AH when preoperative MRI suspected malignancy or written consent could not be obtained. The type of hysterectomy was categorized as either open surgery (i.e., AH) or LH. Kindai University Nara Hospital does not perform LH, opting for AH in all cases. On the other hand, Kindai University Hospital treats approximately all total hysterectomies for uterine fibroids and adenomyosis via LH. Therefore, all LH cases included in our study were performed at Kindai University Hospital, and all AH cases were performed at Kindai University Nara Hospital. (These two hospitals are located in different geographical areas and have different medical specializations.)

Thirty-eight patients who underwent total hysterectomy for uterine fibroids in 2012 (14 AH and 24 LH cases) were included. Of the 14 AH patients, 12 had uterine fibroids and two had adenomyosis. Of the 24 LH patients, 19 had uterine fibroids and six had adenomyosis.

Patient background (age, number of menstrual cycles, body mass index, preoperative symptoms, and rate of previous abdominal surgery) and surgical outcomes (operative time, blood loss, uterine weight removed, postoperative hospital stay, and rate of operative complications) were examined for both AH and LH.

The SF-36 (v. 2.0, Japanese edition) was administered on postoperative day 3; weeks 1, 2, and 4; and months 6 [3, 4]. This questionnaire comprises 36 items and eight subscales, namely physical functioning, physical role (body), bodily pain, general health, vitality, social function (mental), emotional role, and mental health. Subscale scores were calculated and converted using a norm to set the mean value at 50 and the standard deviation at 10 in the Japanese population. In addition, a license agreement for the use of SF-36 was signed with Health Outcomes and Process Evaluation research, and the study was conducted.

### Statistical analysis

Statistical analysis was performed using Student’s *t*-test to compare the mean values between the two groups and the χ^2^ test to compare proportions. A *P*-value < 0.05 was considered statistically significant.

## Results

AH and LH cases are shown in Table 1, and the surgical outcomes are shown in Table 2. No significant differences were found between the AH and LH groups with respect to age, parity, BMI, or rate of previous abdominal surgery. Similarly, no significant differences were found between the two groups in terms of operative time, blood loss, uterine weight, or rate of operative complications. Furthermore, the duration of postoperative hospital stay was significantly shorter in the LH group.

**Table 1:** Patient demographics by procedure

	Laparoscopy hysterectomy (LH) (n = 24)	Abdominal hysterectomy (AH) (n = 14)	*P*-value
Age (year)	43.8 ± 4.3 (37–54)	47.8 ± 7.3 (37–67)	0.07
Parity (time)	2.0 ± 1.0 (0–4)	2.0 ± 1.5 (0–6)	0.92
Body mass index (kg/m^2^)	24.4 ± 3.6 (19.5–33.3)	23.9 ± 3.8 (18.6–32.5)	0.70
Preoperative symptoms	AUB-L 17 Dysmenorrhea 5 Abdominal enlargement 2	AUB-L 9 Dysmenorrhea 3 Abdominal enlargement 2	–
Rate of previous abdominal surgery (%)	20.8 C/S 4 Appendectomy 1	21.4 C/S 2 Appendectomy 1	0.97

*C/S* cesarian section

The top three studies included in Table 1 were conducted using Student’s *t-*test, and the bottom study was conducted using the χ^2^ test

**Table 2:** Surgical outcomes based on procedure

	Laparoscopy hysterectomy (LH) (n = 24)	Abdominal hysterectomy (AH) (n = 14)	*P*-value
Operative time (min)	102 ± 32 (67–192)	99 ± 31 (60–158)	0.80
Blood loss (ml)	242 ± 327 (10–1686)	272 ± 225 (5–755)	0.76
Uterine weight (g)	340 ± 282 (108–1400)	539 ± 367 (90–1342)	0.07
Postoperative hospital stay (day)	3.2 ± 0.5 (3–5)	8.4 ± 0.9 (8–11)	< 0.01
Rate of operative complications (%)	8.3 Hemorrhage 1 Peritonitis 1	7.1 Hemorrhage 1	0.90

In the studies in Table 2, the top four studies were conducted using Student’s *t-*test, and the bottom study was conducted using the χ^2^ test

**Fig. 1:**
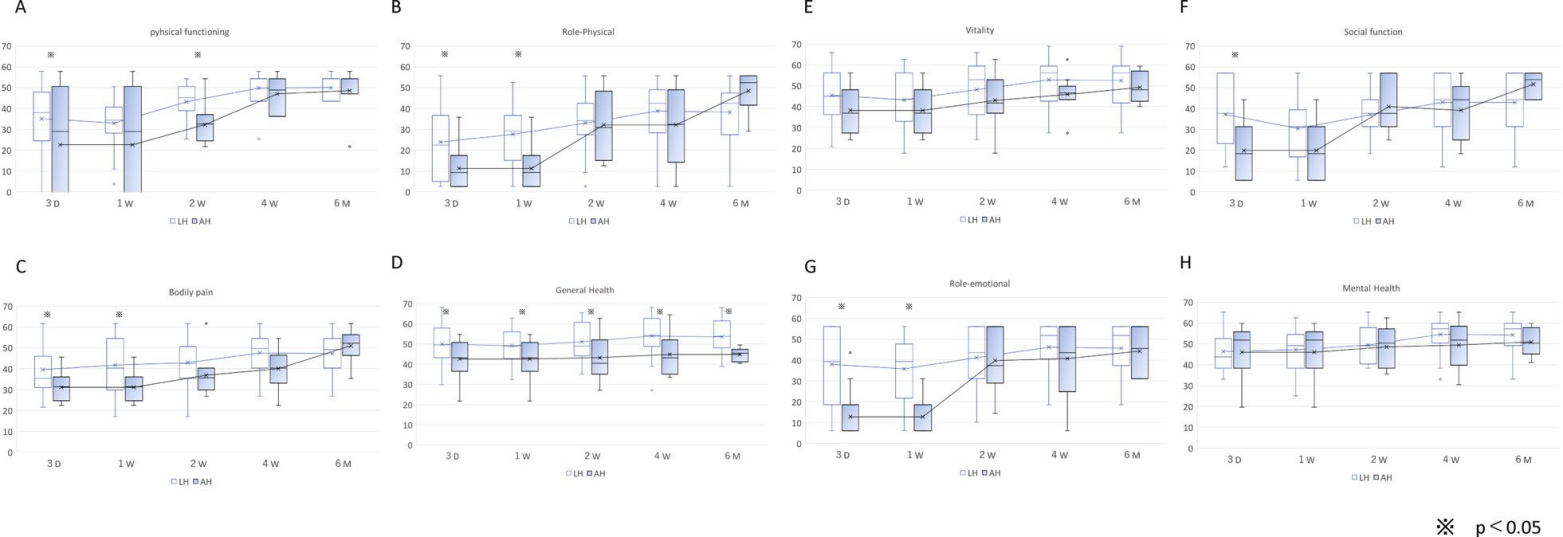
QOL between AH and LH groups. The SF-36 v. 2.0 (Japanese edition) was administered on postoperative day 3; weeks 1, 2, and 4; and month 6. **A** Physical functioning, **B** physical role, **C** bodily pain, **D** general health, **E** vitality, **F** social functioning, **G** emotional role, **H** mental health

Comparisons of the QOL between patients receiving AH or LH are shown in Fig. 1. The LH group had higher scores in physical functioning on postoperative day 3 and week 2; physical role on day 3 and week 1; bodily pain on day 3 and week 1; general health on day 3, weeks 1, 2, and 4, and month 6; social functioning on day 3; and emotional (mental) role on day 3 and week 1. No significant differences were found in overall vitality or mental health.

## Discussion

This is the first report to investigate postoperative QOL using the SF-36 to compare LH and AH for uterine fibroids and adenomyosis. In this context, Andersen et al. [5] conducted a randomized controlled trial comparing open surgery versus laparoscopic surgery for donor nephrectomy using the SF-36 scale as an index of health-related QOL. They reported significantly higher scores in bodily pain and social functioning after laparoscopic surgery. A prospective cohort study of total prostatectomy in Japan used the SF-36 and self-rating of sexual function in postoperative months 1, 3, 6, and 12 and revealed no significant differences between laparoscopic and open surgery [6].

In the field of obstetrics and gynecology, Janda et al. [7] reported postoperative QOL in malignant uterine cancer using the Functional Assessment of Cancer Therapy-General (FACT-G) to compare laparoscopic and abdominal surgery for stage I endometrial cancer. Their study tracked the QOL of cancer patients in the short and long term over a 1-year postoperative period, finding laparoscopic surgery to be advantageous compared with open surgery. Zullo et al. [8] studied the QOL of postoperative patients with endometrial cancer using the SF-36 and reported an overall advantage for laparoscopic surgery in QOL for up to 6 months postoperatively. On the other hand, the Gynecologic Oncology Group (GOG) 2222 (LAP-2) study compared laparoscopic versus abdominal surgery using the FACT-G v. 3.0. Short-term QOL for laparoscopy was significantly superior, but this difference disappeared after 6 months and later for most of the items [9]. Thus, no consensus has been reached on the postoperative QOL in gynecological malignancies. Researchers generally agree on the short-term prognostic advantage of laparoscopy, but opinions vary regarding its long-term prognosis. However, no reports to date have assessed the postoperative QOL for benign diseases.

In addition, the postoperative QOL may vary depending on the number of complications and surgeon experience. Agarwal et al. [10] suggested that the learning curve in performing laparoscopic surgery may be a limiting factor; thus, operative time is likely to decrease as the surgeon’s years of experience increase. In this study, most of the cases were performed by physicians who were in their 10th year of practice for both LH and AH. In addition, Ferrari et al. [11] reported that postoperative management using the enhanced recovery after surgery (ERAS) protocol after obstetrics and gynecology surgery increased the rate of postoperative patient recovery, increased patient satisfaction, and reduced postoperative hospital stay. In this study, the clinical pathway determined that LH cases should be discharged 3–4 days after surgery and after eight days for AH cases; the majority of patients were discharged accordingly. In addition, no special protocol, such as ERAS, was used for postoperative management, and the results were based on standard postoperative management. Therefore, although QOL may be affected by these factors, it remains practically difficult to match all conditions. In this study, the results were obtained from different hospitals, but the conditions were matched to be closer to each other.

Our study revealed that both LH and AH reduce QOL in the immediate postoperative period. Even in this period, however, the QOL was significantly greater in the LH group compared with the AH group. Subsequently, both groups’ scores recovered over time to the national average baseline of 50 points for most variables, and no difference was observed in the long-term QOL between the LH and AH groups in most variables at week 4 and thereafter. Here, the general health scores were significantly higher for LH through 6 months, whereas the mental health and vitality scores showed no significant differences at any time-point. These findings suggest that laparoscopic surgery may be advantageous patients for in the short term regarding physical function and pain but without particular benefits for mental health. Finally, the general health scores were significantly greater for LH at all time points, potentially suggesting that the patients perceive LH as a healthier option.

One of our study’s limitations is the small sample size given the relatively brief study period (i.e., 1 year) and the small proportion of patients who met the eligibility criteria and provided informed consent during this period.

Additionally, the study was conducted in two different facilities with different teams which may have created a bias due to the difference in the study environment of the two groups. The retrospective nature of our study is another limitation; future large-scale prospective studies are encouraged. In addition, the SF-36 used in our study did not include items related to the duration of return to a normal sex life, a metric of importance from the perspective of QOL after hysterectomy.

## Conclusions

The results demonstrate that QOL is increased with laparoscopy in the short postoperative term, improves thereafter in both procedures, and shows no inter-group differences in the long term.

## Data Availability

The datasets used and/or analysed during the current study available from the corresponding author on reasonable request.

## Abbreviations

MRI: Magnetic resonance imaging
LH: Laparoscopic hysterectomy
AH: Abdominal hysterectomy
QOL: Quality of life
SF-36: The Short Form 36-Item Health Survey
FACT-G: The Functional Assessment of Cancer Therapy-General
GOG: The Gynecologic Oncology Group
ERAS: Enhanced recovery after surgery
AUB-L: Abnormal uterine bleeding-Lieomyoma
