# Correction to: Quality of life after laparoscopic hysterectomy versus abdominal hysterectomy

**DOI:** 10.1186/s12905-021-01382-6

**Published:** 2021-06-08

**Authors:** Yasushi Kotani, Kosuke Murakami, Risa Fujishima, Akiko Kanto, Hisamitsu Takaya, Masao Shimaoka, Hidekatsu Nakai, Noriomi Matsumura

**Affiliations:** Department of Obstetrics and Gynecology, Kindai University Faculty of Medicine, 377-2 Ohno-higashi, Osaka-sayama, Osaka 589-8511 Japan

## Correction to: BMC Women’s Health (2021) 21:219 10.1186/s12905-021-01364-8

Following the publication of the original article [1], we were notified that “Research article” was mistakenly included in the title.

The original articles has been updated.
